# Overexpression of *GmNAC03* in Soybean Enhances Salt Tolerance

**DOI:** 10.3390/plants14213235

**Published:** 2025-10-22

**Authors:** Dezhi Han, Wu Zhang, Yang Li, Wei Li, Fanli Meng, Wencheng Lu

**Affiliations:** 1 Heihe Branch of Heilongjiang Academy of Agricultural Sciences, Heihe 164300, China; handezhi2008@163.com (D.H.); guoguo_zw@163.com (W.Z.); liweizjyzc@163.com (W.L.); 2 State Key Laboratory of Black Soils Conservation and Utilization, Northeast Institute of Geography and Agroecology, Chinese Academy of Sciences, Harbin 150081, China; liyang_hrb@iga.ac.cn (Y.L.); mengfanli@iga.ac.cn (F.M.); 3 Key Laboratory of Soybean Molecular Design Breeding, Northeast Institute of Geography and Agroecology, Chinese Academy of Sciences, Harbin 150081, China

**Keywords:** glycine max, salt stress, transcription factor, NAC, RNA-seq

## Abstract

Soybean is a major source of plant-based protein and vegetable oil, but its productivity is severely limited by soil salinity. Transcription factors including NAC family play pivotal roles in regulating stress-responsive pathways. Here, we identified and characterized a salt-induced NAC transcription factor, *GmNAC03*, in soybean. Overexpression of *GmNAC03* significantly improved salt tolerance at both the germination and seedling stages. Physiological analyses revealed that antioxidant enzyme activities, including superoxide dismutase (SOD), peroxidase (POD), and catalase (CAT), were elevated in *GmNAC03* transgenic lines, accompanied by reduced malondialdehyde (MDA) accumulation, indicating enhanced oxidative stress resistance. To further explore its regulatory mechanisms, RNA-seq analysis was performed, which showed that *GmNAC03* overexpression affected pathways related to amino acid metabolism, particularly glutamine and aspartate family amino acid biosynthesis, as well as phenylpropanoid biosynthesis. Differentially expressed genes were enriched in alanine, aspartate, and glutamate metabolism, suggesting a role for *GmNAC03* in metabolic reprogramming under salt stress. Together, these findings demonstrate that *GmNAC03* functions as a positive regulator of salt tolerance in soybean by modulating antioxidant defense and amino acid metabolic pathways. This work provides new insights into the molecular basis of NAC-mediated stress adaptation and offers a potential target for breeding soybean varieties with enhanced salinity resistance.

## 1. Introduction

Soybean (*Glycine max*) is a vital crop for both food and oil production. It serves as a major source of plant-based protein and vegetable oil worldwide [1]. As the global population continues to grow, the demand for food is rising. However, this demand is becoming harder to meet due to both biotic and abiotic stresses, such as salt stress, which limit crop production. Higher soil salinity reduces the number and length of soybean roots, limiting their ability to reach water and nutrients and weakening the transport of essential resources within the plant [2]. Soil salinity poses a threat to crop growth, yield, and long-term agricultural and environmental sustainability. It causes both osmotic and toxic stress in plants, which can slow growth, alter development, affect metabolism, and lead to changes in ions homeostasis [3]. In addition to naturally occurring salinity, soil salinization is increasingly driven by irrigation practices and the impacts of climate change [4]. Global warming, freshwater depletion, improper irrigation methods have all contributed to increasing soil salinity which reduces the amount of arable land and harms the supply of crops vital for human survival [5]. The Food and Agriculture Organization (FAO) reports that over 833 million hectares of soil worldwide are affected by salinity.

Na^+^ is the predominant ion in saline soils, and its excessive accumulation serves as a primary indicator of elevated soil salinity. Excess Na^+^ in saline soil is taken up by plant roots through nonselective cation channels (NSCCs), which facilitate the toxic influx of sodium ions across the plasma membrane [6,7]. Salt stress affects plants in two ways: (1) osmotic stress, which arises from reduced water availability caused by high solute concentrations, and (2) ion toxicity, caused by the excessive accumulation of salts leading to ionic stress [8]. Plants initially encounter water deficit caused by elevated salt ion concentrations and decreased (more negative) osmotic potential in the root zone, which leads to a series of morphological, physiological, biochemical, metabolic, and gene expression changes [9,10].

Because plants cannot move, they have developed a range of effective strategies to handle different environmental stresses [11]. These include controlling salt uptake and transport, sensing and responding to salt, and up-regulating the expression of salt-responsive genes [12]. Gene expression regulation plays a central role in regulating Na^+^ uptake and supports plant adaptation to salt stress by contributing to the maintenance of ion homeostasis. For example, key Na^+^ transporters, including SOS1 (Na^+^/H^+^ antiporter), NHX, and HKT1 (high-affinity K^+^ transporter), are transcriptionally regulated to facilitate Na^+^ efflux and redistribution, thereby alleviating ion toxicity and contributing to osmotic homeostasis [13]. Transcription factors such as NAC, DREB, and MYB regulate the expression of salt-responsive gene expression, thereby enhancing plant salt tolerance [14,15].

Overexpressing salt tolerance-related transcription factors has been proven to be an effective strategy for developing salt-tolerant soybean varieties [16,17]. For example, the overexpression of *GmMYB84* enhances salinity tolerance in soybean [18]. Salt stress reduces DNA methylation levels in the *GmMYB84* promoter, thereby increasing the expression of *GmAKT1* [18]. Overexpression of another transcription factor, *GmbZIP131*, which is phosphorylated by GmSOS2, increases salt resistance in soybean by upregulating the expression of flavonoid biosynthetic genes [19]. Interestingly, the heterologous expression of salt-induced genes from species such as *Arabidopsis thaliana* can also enhance salt tolerance in soybean [17].

NAC transcription factors take part in many plant growth and development processes, including secondary cell wall formation, apical meristem and flower bud differentiation, and lateral root development [20,21]. They also help plants respond to environmental stresses like salt [22]. Several soybean NAC transcription factors, such as *GmNAC06* and *GmNAC81*, have been reported to enhance tolerance to salt stress [23,24]. For example, *GmNAC06* can regulate the expression of downstream genes involved in osmotic adjustment, such as proline and glycine betaine biosynthesis, and maintain ionic homeostasis by modulating Na^+^/K^+^ ratios [23]. GmSIN1 is a salinity-induced NAC transcription factor that functions as a key regulator of soybean salt tolerance. It promotes root growth and enhances salt tolerance by amplifying cellular abscisic acid (ABA) and reactive oxygen species (ROS) levels [25]. Mechanistically, GmSIN1 directly binds to the promoters of *GmNCED3s* and *GmRbohBs*, upregulating ABA biosynthesis and ROS generation, thereby forming a positive feed-forward loop that rapidly transduces and amplifies salt stress signals [25]. In this study, we identified another salt induced NAC transcription factor, *GmNAC03*. Our result suggested that overexpression of *GmNAC03* enhanced soybean salt tolerance. In addition, we performed RNA-seq to investigate the regulatory mechanism underlying the *GmNAC03* transgene. This study offers new insights into the role of *GmNAC03* and paves the way for breeding transgenic soybean varieties with improved salt tolerance.

## 2. Result

### 2.1. Soybean Transformation and Positive Line Identification

RNA-seq analysis of the salt–tolerant soybean cultivar Heihe 60 led to the identification of the transcription factor *GmNAC03* (Glyma.06g248900). The full length of the *GmNAC03* gene is 1014 bp and contains three exons and two introns.

**Figure 1:**
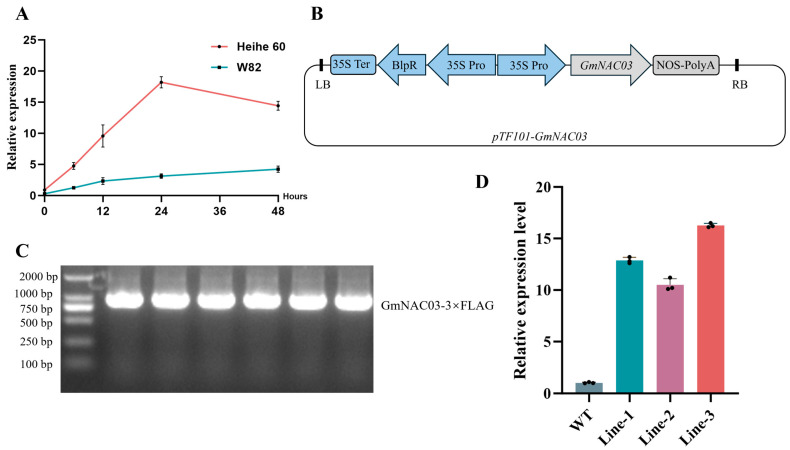
Generation of *GmNAC03* Transgenic Plants. (**A**) RT-qPCR analysis of *GmNAC03* gene expression after salt treatment. (**B**) Schematic illustration of the plant expression construct harboring the *GmNAC03* gene. (**C**) PCR was used to verify six T0-positive transgenic soybean plants. (**D**) RT-qPCR analysis of *GmNAC03* gene expression in three T3 homozygous transgenic lines.

To further examine its expression pattern, we performed an RT-qPCR analysis using both Heihe 60 and W82 soybean seedlings subjected to salt treatment. Salt treatment (200 mM NaCl) was applied at the seedling stage when the primary leaves were fully expanded, and samples were collected from the roots and leaves at 0, 6, 12, 24 and 48 h after treatment. The results showed that *GmNAC03* expression gradually increased and peaked at 24 h after the onset of salt stress (Figure 1A). These findings suggest that *GmNAC03* may play an important role in the soybean response to salt stress.

To gain deeper insight into the stress resistance function of *GmNAC03*, we cloned its coding sequence from the local soybean cultivar Heihe 60 and expressed it under the CaMV 35S promoter (Figure 1B). The wild-type variety, Williams 82 was transformed using *Agrobacterium*-mediated transformation. In total, six T0 transgenic lines were obtained. To verify successful transformation, PCR was performed using a forward primer designed from the vector-specific sequence upstream of the *GmNAC03* insert and a reverse primer located within the *GmNAC03* coding region. The resulting amplicon showed the expected size, confirming successful transformation (Figure 1C). After three generations of selection, three independent homozygous overexpression lines were identified (Lines 1, Lines 2 and Lines 3). RT-qPCR further confirmed that Lines 3 exhibited the highest expression level of *GmNAC03* (Figure 1D).

### 2.2. Overexpression of the GmNAC03 Gene Improves Soybean Salt Tolerance

**Figure 2:**
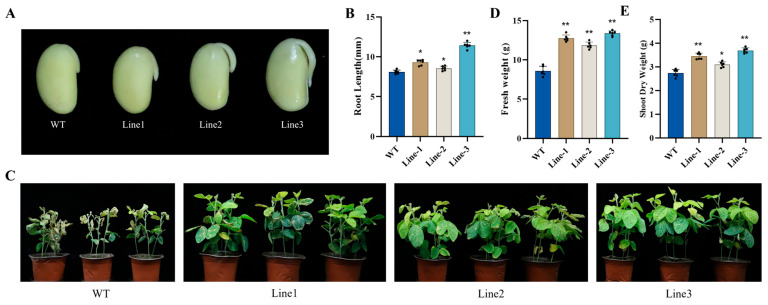
Salt Stress Phenotypes of Transgenic Soybeans and WT. (**A**,**B**) Comparison of phenotypes and root lengths between *GmNAC03* transgenic soybean and wild type under salt stress at the germination stage. (**C**) Phenotypes of *GmNAC03* transgenic soybean and WT under salt treatment during growth stage. (**D**,**E**) Fresh and dry weights of whole plants of *GmNAC03* transgenic soybean and WT under salt treatment during the growth stage. * means *p* < 0.05. ** means *p* < 0.01. The *p* value was calculated by Student’s *t*-test. Each experiment included three biological replicates.

To evaluate the function of *GmNAC03* in soybean, we assessed the salt tolerance of transgenic and wild-type (W82) soybeans at both the germination and vegetative stages. During the germination stage, after a one-week continuous treatment with 200 mM NaCl, the three *GmNAC03* overexpression lines developed longer roots than W82 (Figure 2A,B). Similarly, during the vegetative stage, after a three-week continuous treatment with 200 mM NaCl, W82 plants displayed severe wilting, with drooping, curling leaf margins, and symptoms of chlorosis and dehydration (Figure 2C). In contrast, the *GmNAC03* overexpression lines exhibited significantly less leaf damage, showing reduced chlorosis and marginal necrosis (Figure 2C). Measurements of fresh weight and dry weight of whole plants further confirmed that the transgenic lines experienced less damage than W82 under salt stress (Figure 2D,E). These results suggest that *GmNAC03* overexpression enhances salt tolerance in soybean during both germination and vegetative growth.

### 2.3. Physiological Evaluation of Salt Tolerance

**Figure 3:**
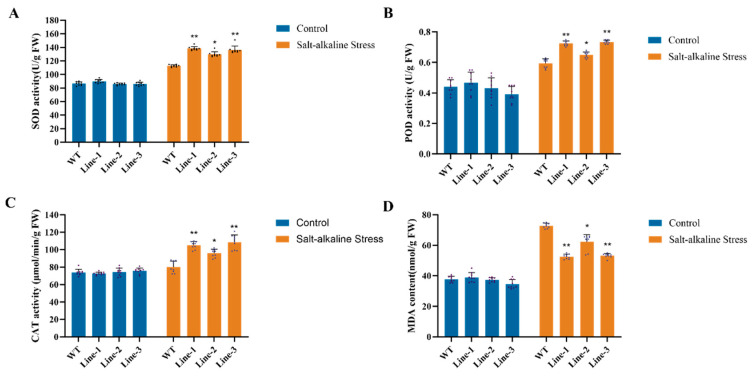
Measurement of physiological parameters in transgenic soybean and WT subjected to salt stress. (**A**) Superoxide dismutase (SOD) activity of *GmNAC03* transgenic soybean and WT before and after exposure to salt treatment. (**B**) Peroxidase (POD) activity of *GmNAC03* transgenic soybean and WT before and after exposure to salt treatment. (**C**) Catalase (CAT) activity of *GmNAC03* transgenic soybean and WT before and after exposure to salt treatment. (**D**) Malondialdehyde (MDA) activity of *GmNAC03* transgenic soybean and WT before and after exposure to salt treatment. * means *p* < 0.05. ** means *p* < 0.01. The *p* value was calculated by Student’s *t*-test. Each experiment included three biological replicates.

To evaluate the salt tolerance of transgenic soybean, we measured the activities of superoxide dismutase (SOD), peroxidase (POD), and catalase (CAT) in both *GmNAC03* overexpression lines and W82 plants under 200 mM NaCl treatment. The activities of all three enzymes increased in both genotypes under salt stress (Figure 3A,C). However, the increase was significantly greater in the transgenic lines than that in W82, suggesting that the transgenic plants have enhanced salt tolerance (Figure 3A,C). In addition, malondialdehyde (MDA) content, an indicator of membrane damage, was significantly higher in W82 than that in the transgenic lines following salt treatment (Figure 3D) Taken together, these results suggest that the transgenic soybean lines suffer less oxidative damage under salt stress compared to W82.

### 2.4. RNA-Seq Analysis of GmNAC03 Overexpression Lines and WT

**Figure 4:**
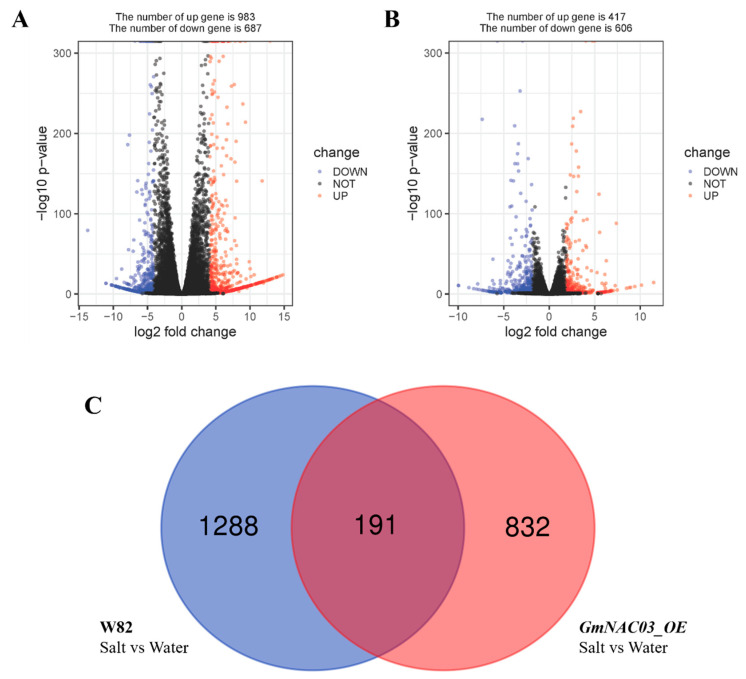
Comparison of differentially expressed genes (DEGs) between transgenic lines and W82 before and after salt treatment. (**A**) Analysis of DEGs in W82 soybean before and after exposure to salt stress. (**B**) Analysis of DEGs in *GmNAC03* overexpression Line 3 before and after exposure to salt stress. (**C**) Venn diagram showing shared and distinct DEGs between W82 and *GmNAC03* overexpression lines.

Next, we performed RNA-seq analysis to identify potential genes regulated by *GmNAC03*. The *GmNAC03* overexpression line (Line 3), which exhibited the highest level of *GmNAC03* expression, and W82 plants were treated with 200 mM NaCl for 24 h and referred to as S-NAC and S-W82, respectively. The same plant materials treated with water were designated as W-NAC and W-W82. The clean reads from each sample were aligned to the Williams 82 reference genome. To assess the quality of the RNA-Seq data, principal component analysis (PCA) was performed using normalized gene expression values. The PCA plot showed high consistency among biological replicates, confirming the reliability of the dataset (Supplementary Figure S1). A total of 1674 differentially expressed genes (DEGs) were identified between S-W82 and W-W82, including 983 up-regulated and 687 down-regulated genes (Figure 4A). Meanwhile, 1023 DEGs were identified between S-NAC and W-NAC, comprising 417 up-regulated and 606 down-regulated genes (Figure 4B). To visualize the expression patterns of DEGs, a heatmap was generated based on normalized expression values of the top 50 highly expressed genes. The heatmap revealed distinct clustering between the control and treatment samples, indicating clear transcriptional differences in response to salt stress, which is consistent with our PCA (Supplementary Figure S2). Then we compared all DEGs to determine the specific genes that are specifically regulated by *GmNAC03*. Interestingly, it was found that the *GmNAC03* overexpression induced 832 unique DEGs upon salt stress (Figure 4C). Furthermore, RT-qPCR was performed to verify the reliability of the RNA-seq data and to exclude possible effects of transgene insertion on the expression of certain genes. This analysis confirmed consistent expression patterns among independent lines and supported the reliability of our conclusions (Supplementary Figure S3). Taken together, these results indicate that overexpression of the *GmNAC03* gene in soybean led to significant alterations in transcriptional profiles in the W82 cultivar, which may contribute to the observed enhancement in salt tolerance.

### 2.5. Gene Ontology Enrichment Analysis of Differentially Expressed Genes

**Figure 5:**
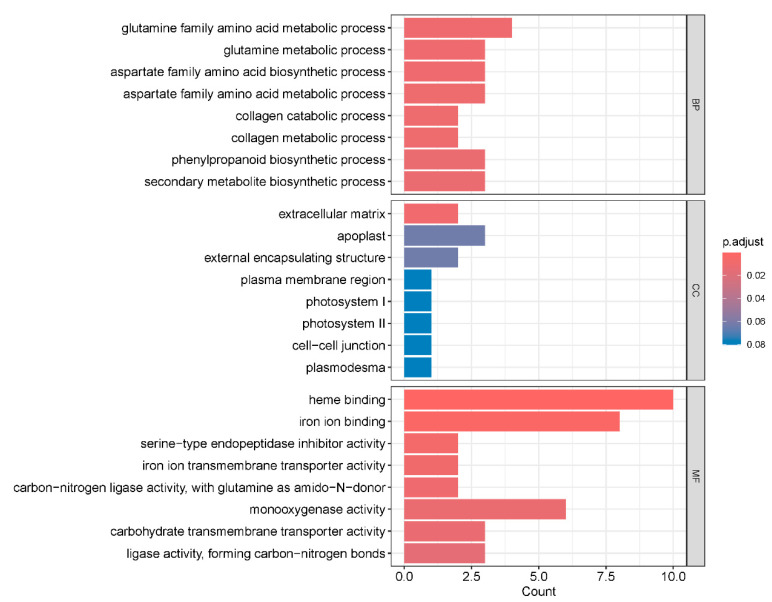
Gene Ontology Enrichment Analysis of *GmNAC03*-specific Differentially Expressed Genes. Analysis of gene ontology for the 832 differentially expressed genes (DEGs) uniquely regulated by *GmNAC03* overexpression.

To investigate the functions of DEGs, we performed gene ontology analysis of DEGs in transgenic soybean and WT plants under salt and water treatments. We focused on the 832 DEGs that were specifically regulated by *GmNAC03* overexpression (Figure 5). Significant gene ontology terms were enriched in biology process such as glutamine family amino acid metabolic process (GO:0009064, *p* = 0.001), aspartate family amino acid biosynthetic process (GO:0009067, *p* = 0.001259) and phenylpropanoid biosynthetic process (GO:0009699, *p* = 0.003679) (Figure 5). On the other hand, for molecular function, significant gene ontology terms were enriched in heme binding (GO:0020037, *p* = 5.25 × 10^−5^), iron ion binding (GO:0005506, *p* = 0.00042) and monooxygenase activity (GO:0004497, *p* = 0.005219) (Figure 5).

### 2.6. Kyoto Encyclopedia of Genes and Genomes Pathway Enrichment Analysis of Differentially Expressed Genes

**Figure 6:**
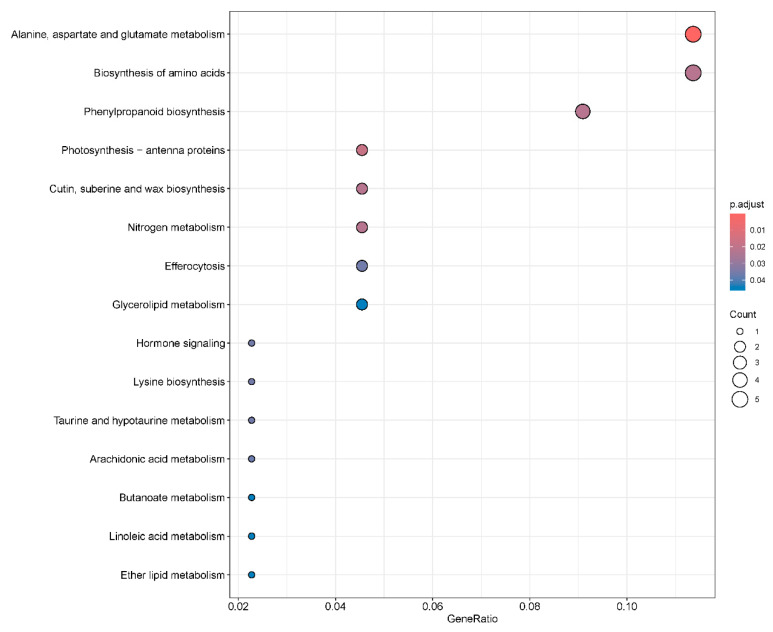
KEGG enrichment analysis of *GmNAC03*-specific DEGs. KEGG analysis for the 832 differentially expressed genes (DEGs) uniquely regulated by *GmNAC03* overexpression. The “GeneRatio” refers to the ratio of the number of differentially expressed genes (DEGs) annotated to a given GO term or KEGG pathway to the total number of DEGs used in the enrichment analysis.

To further investigate the mechanisms underlying the enhanced salt tolerance in *GmNAC03*-overexpressing soybean lines, we performed KEGG pathway analysis on the 832 DEGs specifically regulated by *GmNAC03* (Figure 6). The DEGs were significantly enriched in alanine, aspartate, and glutamate metabolism (gmx00250, *p* = 3.98 × 10^−5^), which was consistent with the GO analysis results, followed by amino acid biosynthesis (gmx01230, *p* = 0.026) and phenylpropanoid biosynthesis (gmx00940, *p* = 0.0319) (Figure 6). Taken together, our results suggest that *GmNAC03* overexpression may enhance soybean salt tolerance by inducing substantial changes in glutamate metabolism.

## 3. Discussion

Salt stress is a major factor limiting soybean growth and yield. In this study, we identified a soybean NAC transcription factor, *GmNAC03*. Overexpression of *GmNAC03* significantly enhances salt tolerance in soybean. RNA-seq analysis indicated that *GmNAC03* perturbs amino acid metabolism, which may be responsible for the increased salt tolerance. Consistently, the Arabidopsis homolog of *GmNAC03*, *AtNAC072*, has also been reported to participate in ABA-mediated stress responses [26,27].

The improved salt tolerance observed in the *GmNAC03*-overexpressing soybean lines can also be explained in light of previously documented morpho-physiological reactions to salinity stress, in addition to the transcriptional regulation identified in this investigation. When compared to sensitive cultivars, salt-tolerant soybean cultivars often maintain superior growth performance and biomass accumulation, especially through prolonged root development and postponed leaf senescence [28,29].

Also, these tolerant genotypes have a more stable Na^+^/K^+^ ratio and less buildup of Na^+^ and Cl^+^, both of which are necessary to keep cells intact and ions in balance when the salt level is high [30]. Furthermore, numerous studies have demonstrated that elevated antioxidant enzyme activities, such as those of superoxide dismutase (SOD), peroxidase (POD), and catalase (CAT), aid in detoxifying reactive oxygen species (ROS) and protect against oxidative membrane damage [31,32]. During salt stress, the accumulation of suitable solutes such proline is also essential for osmotic adjustment and membrane stability [33].

Consistent with our findings, the importance of the glutamine family amino acid metabolic process identified in the GO analysis has also been highlighted in previous studies. For example, a transcriptome analysis in *Medicago truncatula*, a closely related species, reported that the glutamine family amino acid metabolic process was enriched among the DEGs under alkaline stress [34]. In the KEGG pathway analysis, the DEGs were enriched in phenylpropanoid biosynthesis, which represents a core metabolic route for the production of isoflavones in plants. Isoflavones are well known to enhance plant resistance by reducing ROS-induced damage and maintaining membrane integrity. In legumes, they also function as signaling molecules that regulate the expression of defense-related genes. Additionally, the DEGs were enriched in cutin, suberin, and wax biosynthesis pathways, consistent with previous research showing that this pathway is enriched among the DEGs of yardlong bean under salt stress [35]. The accumulation of these compounds thickens and strengthens the epidermal and endodermal layers, thereby reducing the penetration of toxic ions such as Na^+^ and Cl^−^ under saline–alkaline conditions.

Salt stress significantly impacts soybean growth and yield. Transgenic breeding has emerged as an effective approach for developing cultivars with improved salt tolerance. For example, overexpression *GmMYB84* confers salinity tolerance in soybean [18]. Interestingly, it was reported that heterologous expression of salt-induced genes from other plant species also enhances soybean salt tolerance. For example, the heterologous expression of the *AtARA6* gene from *Arabidopsis thaliana* confers enhanced salt tolerance on soybean by promoting the expression of vesicle transport-related genes [16]. However, many studies, including ours, lack an in-depth understanding of gene function. How the overexpressed transcription factor of interest regulates its target genes remains to be explored. Transcriptome sequencing, combined with advanced techniques such as ChIP-seq (Chromatin Immunoprecipitation Sequencing) and DAP-seq (DNA Affinity Purification Sequencing), may facilitate the identification of target genes.

Although recent advances have deepened our understanding, key gaps and controversies remain, including the stability of epigenetic memory, the balance between stress tolerance and growth, hormonal crosstalk, and the roles of novel genes in salt tolerance. Resolving these challenges will require continued research using tools such as GWAS, transcriptomics, transgenic and genome-editing technologies, alongside studies on energy allocation and hormonal control. A more in-depth examination of these complex and synergistic mechanisms will contribute to improving plant resilience and facilitating adaptation to increasingly adverse environmental conditions.

## 4. Materials and Methods

### 4.1. Plant Materials and Growth Conditions

Unless otherwise noted, *GmNAC03* overexpression lines and recipient soybean W82 were grown at 25 °C in a greenhouse under long days condition (16 h/8 h light/dark).

### 4.2. Vector Construction

Total RNA was extracted from Heihe 60 roots and reverse-transcribed into cDNA. The *GmNAC03* gene (Glyma.06g248900) was amplified using specific primers (Supplemental Table S1). In parallel, the pTF101 vector was digested with XbaI and BamHI (NEB). The resulting PCR product was then ligated into the digested vector, hereafter referred to as pTF101-*GmNAC03*.

### 4.3. Genetic Transformation and Progeny Identification in Soybean

The pTF101-*GmNAC03* construct was introduced into *Agrobacterium tumefaciens* strain EHA101. Transgenic soybean plants harboring the construct were regenerated following previously described protocols [16,36]. Genomic DNA was extracted from the progeny of regenerated plants, and PCR-positive individuals were identified using specific primers (Supplemental Table S1).

### 4.4. Salt Stress Treatment

The *GmNAC03* overexpression lines and W82 soybean seeds were sown in vermiculite and irrigated with the 200 mM NaCl solution [37]. Root length was measured after 1 week. For salt stress treatment at the emergence stage, NaCl concentrations were selected according to the previous published protocol slight modifications [38]. The seeds were first sown in vermiculite and watered for 12 days until the primary leaves had fully expanded, after which they were treated with 200 mL NaCl (200 mM). The salt solution was replaced every 3 days. Phenotypic observations were made 1, 2, and 3 weeks after the onset of treatment.

### 4.5. Determination of Salt Tolerance Parameters

Representative physiological salt tolerance indicators in *GmNAC03* overexpression lines and W82, including superoxide dismutase (SOD), peroxidase (POD), and catalase (CAT) activities, were measured after 3 weeks of salt treatment.

SOD activity was determined based on the photochemical reduction of p-nitroblue tetrazolium chloride (NBT), with absorbance recorded at 560 nm [39]. Briefly, the reaction mixture (3 mL total volume) contained 50 mM phosphate buffer (pH 7.8), 13 mM methionine, 75 μM NBT, 0.1 mM EDTA-Na_2_, and 20 μM riboflavin. The reaction was initiated by exposing the mixture to light at 4000 lux for 30 min at 25 °C, while the control was kept in the dark. After illumination, the absorbance was recorded at 560 nm using a spectrophotometer, with the dark sample serving as the reference. One unit of SOD activity (U) was defined as the amount of enzyme required to inhibit 50% of the NBT photoreduction under the assay conditions. Enzyme activity was expressed as units per gram fresh weight (U g^−1^ FW h^−1^).

POD activity was measured using guaiacol as a substrate, with enzyme activity calculated from the reaction rate per unit time [40]. Briefly, the reaction mixture (3 mL) consisted of 50 mM phosphate buffer (pH 6.0), 28 μL guaiacol, and 19 μL 30% H_2_O_2_. The reaction was started by adding 20 μL of enzyme extract, and absorbance was measured every minute for 3 min at 470 nm. One unit of POD activity (U) was defined as the amount of enzyme that caused an increase of 0.01 in absorbance per minute under the assay conditions. Enzyme activity was expressed as ΔA470 min^−1^ g^−1^ FW.

CAT activity was determined by monitoring the decrease in absorbance at 240 nm during H_2_O_2_ decomposition. Briefly, the reaction mixture (2.5 mL) contained 0.1 M phosphate buffer (pH 7.0) and 0.1 M H_2_O_2_ in a ratio of 4:1. The reaction was initiated by adding 0.05 mL of enzyme extract. The decrease in absorbance at 240 nm was recorded every minute for 3 min using a quartz cuvette. One unit of CAT activity (U) was defined as the amount of enzyme required to decrease the absorbance by 0.01 per minute under the assay conditions. Enzyme activity was expressed as ΔA240 min^−1^ g^−1^ FW.

### 4.6. RT-qPCR Validation of Gene Expression

Plant samples collected at different time points after salt treatment were used for qPCR validation. Total RNA was extracted using the FastPure Cell/Tissue Total RNA Isolation Kit (Vazyme, Hopkinton, MA, USA, RC112-01), and genomic DNA was removed using a gDNA removal mix. First-strand cDNA was synthesized with the HiScript IV RT SuperMix (Vazyme, R423-01) according to the manufacturer’s instructions. The synthesized cDNA was then used as the template for qPCR with the SYBR Green Master Mix. Quantitative PCR was performed using the RT-qPCR SYBR Green Kit (Vazyme, Q226-01) with fluorescence-based detection. *GmActin* was used as the internal reference gene. Three biological replicates were included for each treatment. RT-qPCR was carried out on a CFX Opus 96 Dx Real-Time PCR System (Bio-Rad, Hercules, CA, USA), and relative expression levels were calculated using the 2^−ΔΔCT^ method.

### 4.7. RNA-seq Analysis

RNA-seq analysis was performed using *GmNAC03* overexpression line 3 and Williams 82 (W82) as the control. Soybean plants were grown in greenhouse for 7 d and then treated with 200 mM NaCl for 24 h. Whole seedlings, including both shoots and roots, were used for RNA extraction and transcriptome analysis. Total RNA was extracted using the FastPure Cell/Tissue Total RNA Isolation Kit (Vazyme, RC112-01). Three independent biological replicates were used for each treatment group. Library construction and transcriptome sequencing were carried out by Novogene Co., Ltd. (Beijing, China). Briefly, The RNA libraries were sequenced on the Illumina NovaSeq 6000 platform (Illumina, San Diego, CA, USA) to generate 150 bp paired-end reads.

The quality of the raw RNA-seq reads was assessed using FastQC software (version 0.11.9) [41]. Adapter sequences and low-quality bases were trimmed using Trim Galore (version 0.6.7). Clean reads were aligned to the soybean reference genome (Glycine_max_v4.0, NCBI) using HISAT2 (version 2.2.1) [42]. On average, each sample produced approximately 3-4 Gb of clean data, with more than 95% of reads successfully mapped to the Glycine max reference genome (Wm82.a4.v1). Read quantification was performed with featureCounts (version 2.0.1) [43]. Normalization of read counts and identification of differentially expressed genes (DEGs) between sample groups were conducted using DESeq2 (version 1.36.0) [44]. The DEGs were determined based on two criteria: an adjusted *p*-value (FDR) < 0.05 for statistical significance, and a minimum effect size determined by |log_2_Fold Change| ≥ (μ + 2σ), where μ and σ are the mean and standard deviation of the absolute log_2_ fold changes across all genes, respectively. The resulting DEGs were used for subsequent functional enrichment analyses.

### 4.8. Gene Ontology and KEGG Pathway Analysis

The R package AnnotationHub (version 3.6.0) was used to retrieve soybean genome annotation files from the public database. Differentially expressed gene (DEG) lists, separated into up-regulated and down-regulated groups, were subjected to Gene Ontology (GO) enrichment analysis using the R package clusterProfiler (version 4.6.0). KEGG pathway enrichment analysis was also performed with clusterProfiler to systematically identify the metabolic pathways associated with the DEGs and to infer their potential biological functions.

## Data Availability

The original contributions presented in this study are included in the article and Supplementary Materials. Further inquiries can be directed to the corresponding author.

## Supplementary Materials

The following supporting information can be downloaded at https://www.mdpi.com/article/10.3390/plants14213235/s1, Figure S1: Principal component analysis (PCA) of RNA-seq data. Principal component analysis (PCA) was performed based on normalized gene expression values from all samples. The plot shows clear separation between control and treatment groups, indicating distinct transcriptional profiles. Biological replicates clustered closely together, confirming the reliability and consistency of the RNA-seq data; Figure S2: Heatmap showing the expression patterns of DEGs. A heatmap was generated based on normalized expression values of the top 50 highly expressed DEGs. The color scale represents relative expression levels across samples. The heatmap shows clear clustering between control and salt-treated groups, indicating distinct transcriptional responses to salt stress, which is consistent with the PCA results. Figure S3: Validation of RNA-seq results by RT-qPCR. RT-qPCR analysis was conducted to verify the reliability of the RNA-seq data and to assess whether transgene insertion affected gene expression. One upregulated gene (GmWRKY1, Glyma.16G164200) and one downregulated gene (GmPRX4, Glyma.04G052000) were selected for validation. The expression trends obtained by RT-qPCR were consistent with those from RNA-seq, confirming the robustness of the transcriptomic data. Table S1. Sequences of primers used in this study.
