# Supplementary material for: Overexpression of GmNAC03 in Soybean Enhances Salt Tolerance
Source: Plants (Basel). 2025 Oct 22;14(21):3235. doi: 10.3390/plants14213235 (PMC12609559; doi:10.3390/plants14213235)
Supplement: Supplementary file 1 [file plants-14-03235-s001.zip › plants-3866606-supplementary figures.pdf]

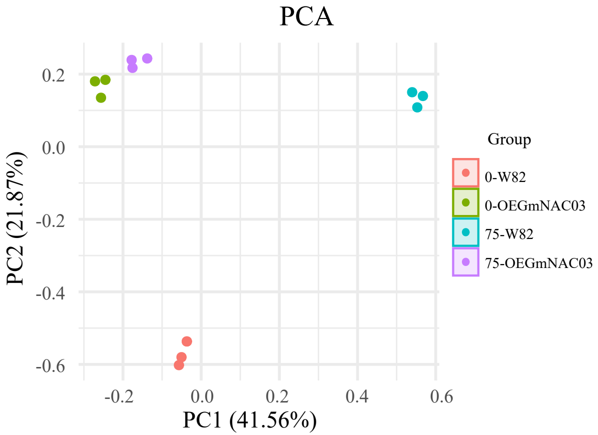


**Supplementary Figure S1. Principal component analysis (PCA) of RNA-seq data.**
Principal component analysis (PCA) was performed based on normalized gene expression values from all samples. The plot shows clear separation between control and treatment groups, indicating distinct transcriptional profiles. Biological replicates clustered closely together, confirming the reliability and consistency of the RNA-seq data.

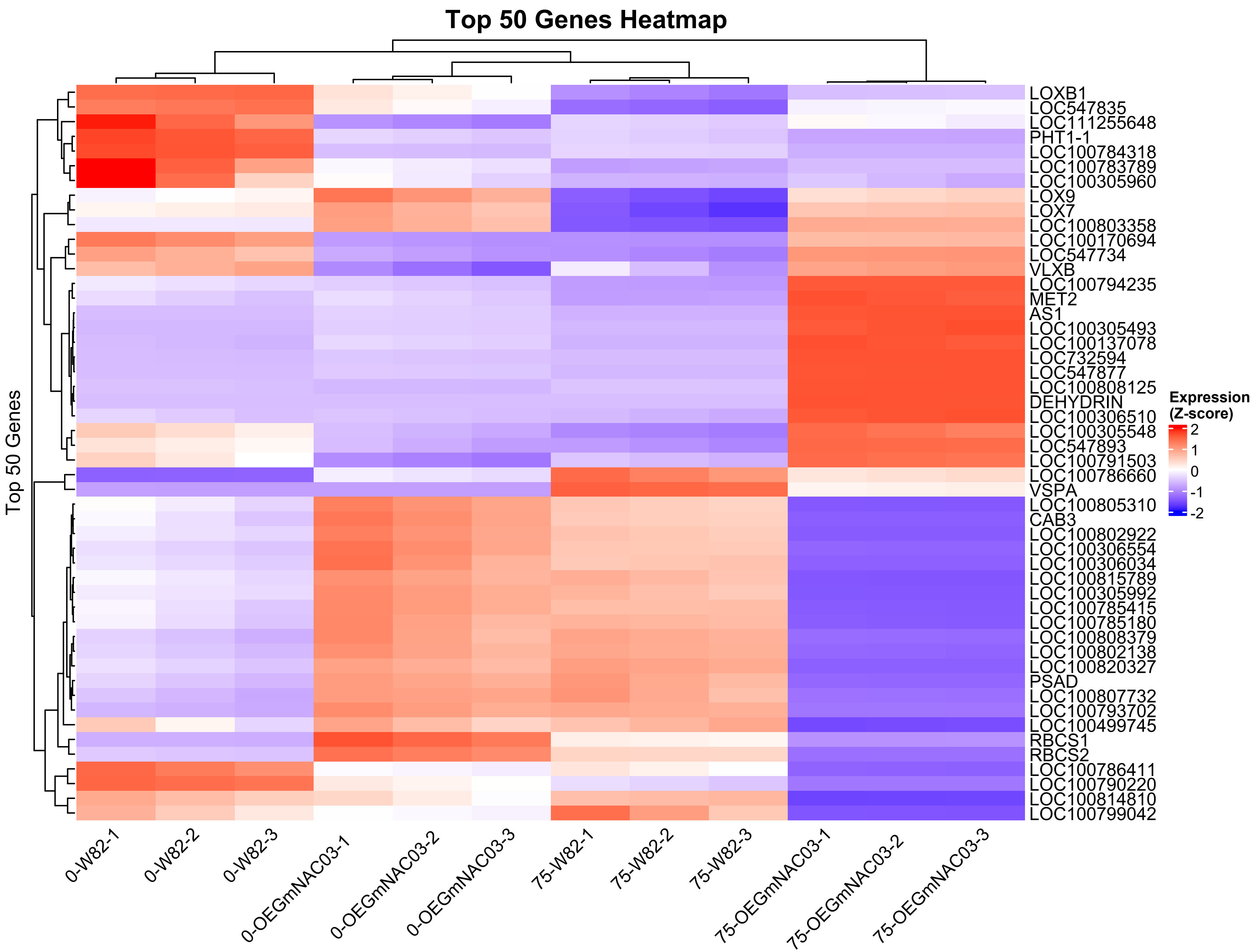


**Supplementary Figure S2. Heatmap showing the expression patterns of DEGs.**
A heatmap was generated based on normalized expression values of the top 50 highly expressed DEGs. The color scale represents relative expression levels across samples. The heatmap shows clear clustering between control and salt-treated groups, indicating distinct transcriptional responses to salt stress, which is consistent with the PCA results.

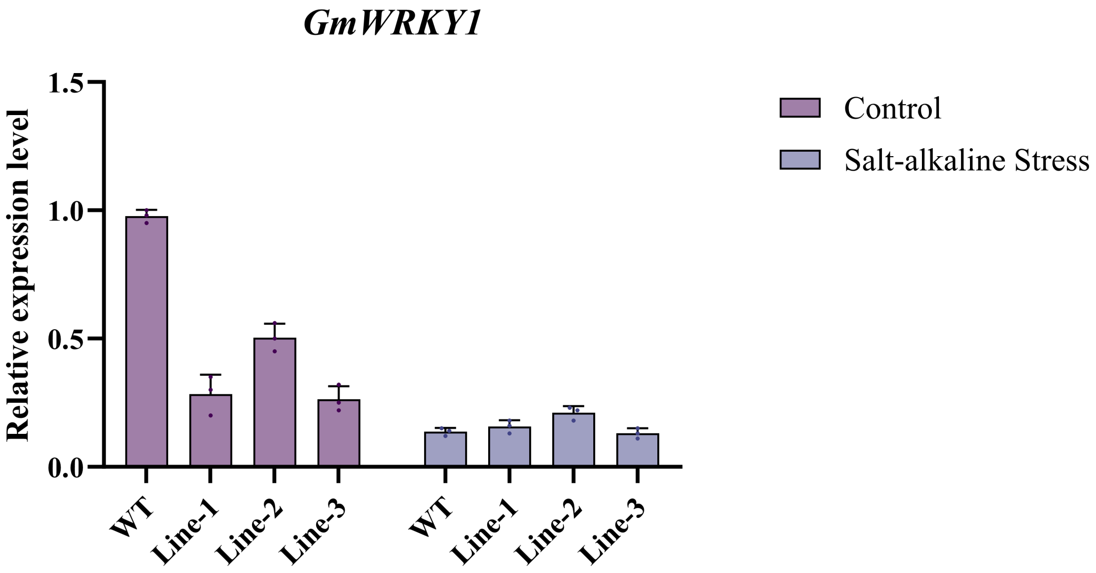


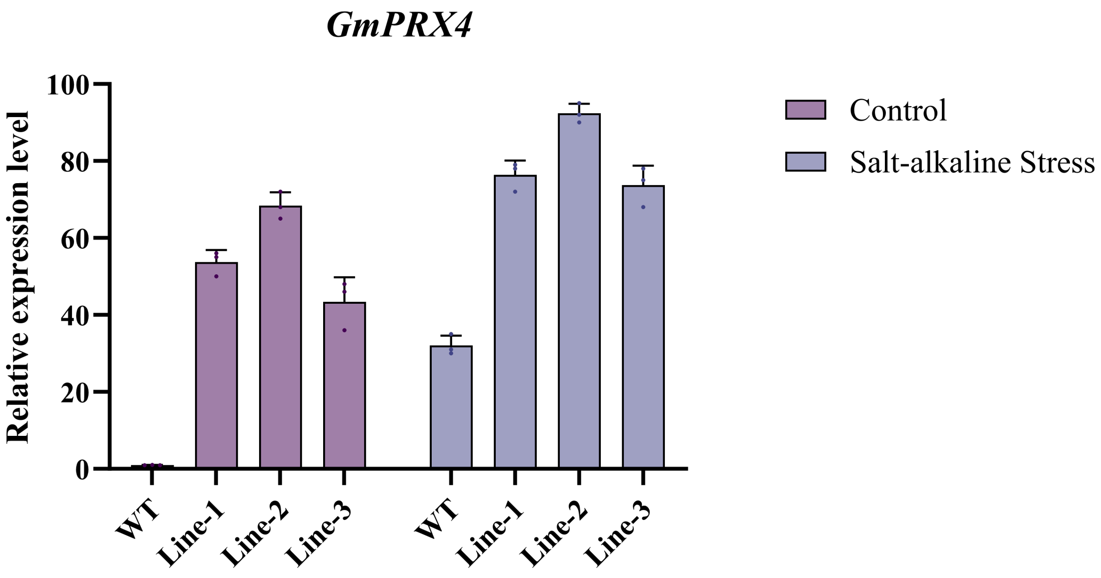


**Supplementary Figure S3. Validation of RNA-seq results by RT-qPCR.**
RT-qPCR analysis was conducted to verify the reliability of the RNA-seq data and to assess whether transgene insertion affected gene expression. One upregulated gene (*GmWRKY1,* Glyma.16G164200) and one downregulated gene (*GmPRX4,* Glyma.04G052000) were selected for validation. The expression trends obtained by RT-qPCR were consistent with those from RNA-seq, confirming the robustness of the transcriptomic data.
